# Inhibition of GTRAP3-18 May Increase Neuroprotective Glutathione (GSH) Synthesis

**DOI:** 10.3390/ijms130912017

**Published:** 2012-09-20

**Authors:** Koji Aoyama, Toshio Nakaki

**Affiliations:** Department of Pharmacology, Teikyo University School of Medicine, 2-11-1 Kaga, Itabashi, Tokyo 173-8605, Japan; E-Mail: kaoyama@med.teikyo-u.ac.jp

**Keywords:** glutathione, cysteine uptake, GTRAP3-18, EAAC1, neurodegeneration

## Abstract

Glutathione (GSH) is a tripeptide consisting of glutamate, cysteine, and glycine; it has a variety of functions in the central nervous system. Brain GSH depletion is considered a preclinical sign in age-related neurodegenerative diseases, and it promotes the subsequent processes toward neurotoxicity. A neuroprotective mechanism accomplished by increasing GSH synthesis could be a promising approach in the treatment of neurodegenerative diseases. In neurons, cysteine is the rate-limiting substrate for GSH synthesis. Excitatory amino acid carrier 1 (EAAC1) is a neuronal cysteine/glutamate transporter in the brain. EAAC1 translocation to the plasma membrane promotes cysteine uptake, leading to GSH synthesis, while being negatively regulated by glutamate transport associated protein 3-18 (GTRAP3-18). Our recent studies have suggested GTRAP3-18 as an inhibitory factor for neuronal GSH synthesis. Inhibiting GTRAP3-18 function is an endogenous mechanism to increase neuron-specific GSH synthesis in the brain. This review gives an overview of EAAC1-mediated GSH synthesis, and its regulatory mechanisms by GTRAP3-18 in the brain, and a potential approach against neurodegeneration.

## 1. Introduction

The brain is vulnerable to oxidative stress because of its high demand for oxygen, abundant unsaturated fatty acids that are targets of lipid peroxidation, and lower antioxidant enzyme activities compared to other organs [1,2]. Under normal physiological conditions, antioxidant mechanisms function efficiently in the brain to overwhelm the lethal insults to neurons by reactive oxygen species (ROS). However, disequilibrium due to increased ROS production or decreased antioxidant defense systems would cause oxidative stress, which is considered a causal factor for neurodegeneration [3–5]. Glutathione (GSH) is the most abundant thiol-containing molecule and one of the most important substances in the brain for neuroprotection. GSH is a tripeptide synthesized from glutamate, cysteine and glycine via two enzymatic steps. Previous studies focused on the enzymatic regulation of GSH synthesis, and the regulatory mechanisms for the neuronal transport system of the rate-limiting substrate, cysteine, have not been clarified. Our recent studies have helped reveal the regulatory mechanisms for cysteine uptake leading to neuronal GSH synthesis. In the present review, we provide an overview of the key molecular mechanisms underlying cysteine uptake leading to increase neuronal GSH content in the brain.

## 2. Glutathione as an Antioxidant

**Figure 1:**
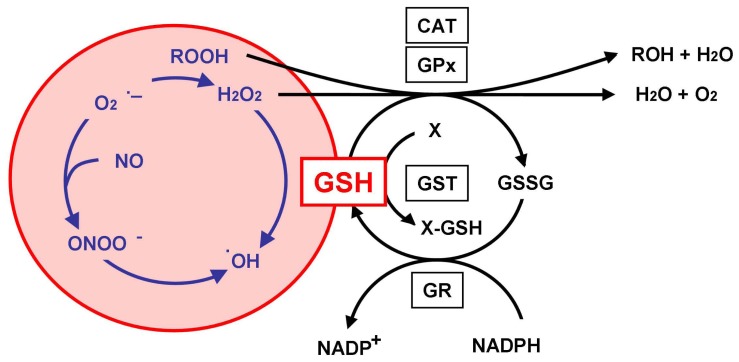
Glutathione (GSH) as an antioxidant. Hydrogen peroxide (H_2_O_2_) and hydroperoxides (ROOH) are degraded by GSH peroxidase (GPx) to water and alcohols, respectively. GSH disulfide (GSSG), the oxidized form of GSH, is reduced back to GSH by the reaction of GSH reductase (GR) with NADPH. Catalase can remove H_2_O_2_ but not ROOH under normal physiological conditions. GSH conjugates with various endogenous and xenobiotic compounds (X), mediated by GSH-*S*-transferase (GST) to remove X from the cell. GSH can also react non-enzymatically with superoxide (O_2_^•−^), nitric oxide (NO), hydroxyl radical (^•^OH), and peroxynitrite (ONOO^−^).

GSH reacts non-enzymatically with superoxide, nitric oxide, hydroxyl radical, and peroxynitrite as an antioxidant [6] (Figure 1). Superoxide is generated by mitochondria in the process of ATP production. Superoxide or nitric oxide *per se* are not toxic unless they react with each other to form peroxynitrite [7], which is a potent oxidant in the brain [8]. Hydroxyl radicals form another potent oxidant, produced from hydrogen peroxide (H_2_O_2_) via the Fenton reaction or peroxynitrite decomposition [7,9], although the reaction rate of hydroxyl radical production is slow and the diffusion distance of hydroxyl radical is limited to much less than that of peroxynitrite [10]. GSH reacts directly with these oxidants to inhibit oxidative stress in the cell. In addition, GSH reacts enzymatically with GSH peroxidase (GPx) and GSH-*S*-transferase (GST) against neurodegeneration [6]. GPx requires GSH as electron donor to react with H_2_O_2_, which is produced from superoxide catalyzed by superoxide dismutase, or endogenous hydroperoxides, which are formed by lipid peroxidation [11]. In the process of peroxide disposal, GSH is oxidized to GSH disulfide (GSSG), which is then reduced back to GSH by GSH reductase (GR) with NADPH (nicotinamide adenine dinucleotide phosphate-oxidase) [12]. In neurons, GR is sufficiently active to allow the quick reduction of the accumulated GSSG [13]. GST reacts with various xenobiotics to form GSH conjugation, leading to detoxication of the compounds and their excretion from the cell.

GSH is resistant to intracellular degradation; it is hydrolyzed solely by γ-glutamyltranspeptidase extracellularly to form γ-glutamyl moiety and a cysteinyl-glycine conjugate, which is then cleaved by dipeptidase to generate cysteine and glycine. GSH conjugation mediated by GST is a major cause of intracellular GSH depletion. Another cause of intracellular GSH depletion is due to thiol-disulfide formation from two GSH molecules to GSSG. Under normal conditions, the intracellular GSSG level is low, and the GSH/GSSG ratio in tissues is high (99:1) [14]. Under insulted conditions, the GSH/GSSG ratio would be 49:1 [15]. Under oxidative stress, GR maintains the equilibrium of the GSH/GSSG redox state in the cell. GSH can also reversibly react with protein thiol groups, which are important for the protein functions as enzymes or receptors, to prevent irreversible protein oxidation [16]. Severe oxidative stress leads to the excessive production of GSSG, overwhelming the reduction of GSSG back to GSH. The excessive GSSG is then exported outside the cell to decrease the intracellular GSH level. Importantly, GSH depletion induced by oxidative stress can subsequently exacerbate oxidative injury in the brain. However, it has been debated whether oxidative stress consistently precedes GSH depletion in patients with neurodegenerative diseases.

## 3. Glutathione Depletion and Neurodegeneration

The GSH level in the brain is approx. 2–3 mM [17]. However, the GSH content varies among brain regions and cell types. The GSH level is the highest in the cortex, followed by the cerebellum, hippocampus, and striatum, and it is lowest in the substantia nigra [18]. Importantly, the basal GSH level is lower in neurons than in glial cells [19], suggesting different mechanisms for preserving GSH homeostasis between neuron and glial cells. In addition, GPx activity is lower in neurons than in glial cells [13,19,20]. Certainly, the ability to detoxify peroxides is less efficient in neurons than in astrocytes [13,19]. Catalase, as is the case for GPx, can also detoxify H_2_O_2_ via degradation to H_2_O and O_2_. However, neuronal catalase activity does not compensate for the peroxide detoxication [19]. Catalase can remove only H_2_O_2_, but not hydroperoxides [21]. These results indicate that neurons are more vulnerable to oxidative stress than are glial cells in the brain, leading to neurodegeneration.

Previous articles indicate an involvement of GSH depletion in neurodegeneration [2,22–24]. Indeed, some neurodegenerative diseases have shown decreased GSH levels in the brain [25,26], but it remains unclear whether the decreased GSH level is a cause or an outcome of neurodegeneration. In patients with Parkinson’s disease (PD), the GSH level was found to be significantly lower than that of age-matched controls [25], whereas the levels of other antioxidants, such as ascorbate and α-tocopherol, were unchanged in the substantia nigra [27] and basal ganglia [28], respectively. Postmortem analysis revealed that normal individuals with incidental Lewy bodies—who would be considered pre-symptomatic PD subjects—showed decreased GSH levels in the substantia nigra compared to those of age-matched controls without Lewy bodies [29]. However, these pre-symptomatic PD subjects did not show any difference in the levels of pro-oxidants, such as iron, ferritin, copper, manganese, and zinc, or mitochondrial enzyme activities in the substantia nigra. These results strongly suggest that GSH depletion is the primary cause of neurodegeneration, and not only a result of oxidative stress [30,31].

## 4. Approaches to Increase the GSH Levels in the Brain

### 4.1. Cysteine Uptake

**Figure 2:**
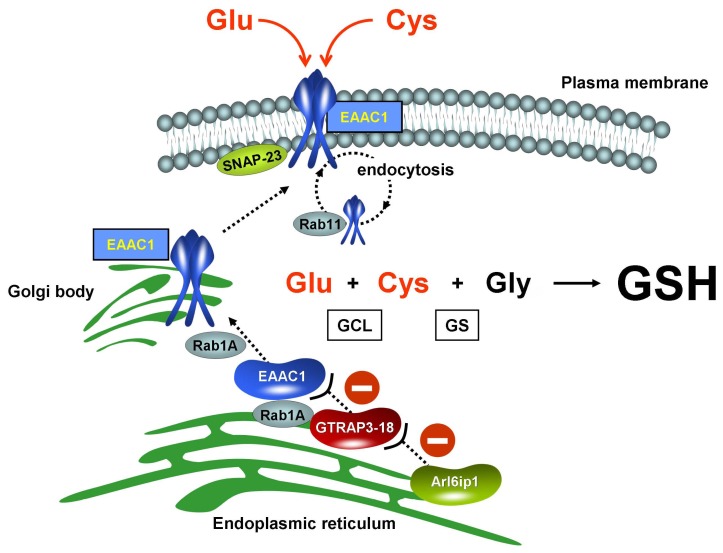
EAAC1/GTRAP3-18-mediated GSH synthesis in neurons. GSH is produced from three amino acids, *i.e.*, glutamate (Glu), cysteine (Cys), and glycine (Gly) by reactions with two enzymes, γ-glutamylcysteine ligase (GCL) and GSH synthase (GS). Translocated to the plasma membrane, EAAC1 transports glutamate and cysteine into the neuron to increase GSH synthesis. Glutamate transporters, as the functioning forms, are present predominantly as trimers on the plasma membrane. SNAP-23 facilitates EAAC1 expression on the plasma membrane. EAAC1 is subject to internalization into early endosome and is recycled back to the plasma membrane in a Rab11-dependent manner. Rab1A regulates multiple membrane trafficking pathways. GTRAP3-18, a protein in the endoplasmic reticulum (ER), binds to Rab1A to interfere with the ER-Golgi transport of EAAC1. Moreover, GTRAP3-18 directly interacts with EAAC1 and retains EAAC1 at the ER to inhibit neuronal GSH synthesis. Arl6ip1 is a GTRAP3-18 interacting protein leading to decrease in GTRAP3-18/EAAC1 interaction. Arl6ip1 positively modulates EAAC1-mediated glutamate transport.

GSH synthesis consists of two consecutive enzymatic steps requiring ATP (Figure 2). The first step is the reaction of glutamate with cysteine catalyzed by glutamate cysteine ligase (GCL), and the second is the reaction of γ-glutamylcysteine with glycine catalyzed by GSH synthase (GS). In neurons, the availability of cysteine, but not that of glutamate or glycine, limits intracellular GSH levels [32]. Compared to the intracellular concentrations of glutamate and glycine, the cysteine concentration is maintained at much lower levels because of its toxicity to the cell [33–36]. The first step, *i.e.*, the catalysis by GCL, is considered the rate-limiting reaction for GSH synthesis. The Km value of GCL for cysteine is 0.1–0.3 mM: that of glutamate is 1.8 mM [37]. The intracellular glutamate concentration is much higher than the Km value, whereas the intracellular cysteine level is around the Km value. Therefore, the intracellular cysteine level is considered the rate-limiting precursor for GSH synthesis.

### 4.2. EAAC1 for Neuronal Cysteine Uptake

In neurons, cysteine uptake is mediated mainly by one of the Na^+^-dependent excitatory amino acid transporters, named excitatory amino acid carrier 1 (EAAC1) (Figure 2). EAAC1 is present in both glutamatergic and GABAergic neurons [38]. A number of studies have demonstrated that oxidative stress is involved in glutamate-induced neurotoxicity. Free radicals can increase the glutamate level in the synaptic cleft by increasing glutamate release and decreasing glutamate re-uptake [39]. Neuronal NMDA receptor activation leads to the formation of superoxide, causing oxidative stress [40]. EAAC1 was first reported as a neuronal glutamate transporter, although most of the glutamate uptake in the brain is dependent on transport by the glial transporters, such as GLAST and GLT-1 [41–43].

Notably, EAAC1 can also transport cysteine, the rate-limiting substrate for GSH synthesis, at a rate comparable to that of glutamate, whereas GLAST and GLT-1 can preferentially transport glutamate [44]. A previous study demonstrated that the glutamate transport was abolished in the R447C mutant of EAAC1, whereas that of cysteine was conserved, suggesting a different cysteine transport system from that of glutamate [45]. A recent study showed that the [^3^H]glutamate uptake activity in proteoliposomes prepared from GLT-1 knockout mouse brain was quite low (~2% of wild-type) compared to that from the wild-type, suggesting that the contribution of EAAC1 to synaptic glutamate clearance is almost negligible [38]. Indeed, the EAAC1 density in the rat hippocampus was 100 times lower than that of GLT-1 [38]. EAAC1 is observed in neuronal soma and dendrites, but not in synaptic terminals [38,46,47]. These results support the hypothesis that the main role of EAAC1 in the brain is not neurotransmission but neuronal metabolism related to cysteine uptake.

### 4.3. Regulation of EAAC1 Translocation to the Plasma Membrane

The expression of EAAC1 on the plasma membrane is only 20% of the total intracellular EAAC1 under normal conditions [48]. Glutamate transporters, including EAAC1, are predominantly present as trimers on the plasma membrane [42]. Translocated to the plasma membrane, EAAC1 can transport glutamate and cysteine for GSH synthesis. EAAC1 is translocated to the plasma membrane by phosphoinositide 3-kinase or protein kinase C (PKC) activation. Although EAAC1 is subject to a transcriptional regulation by nuclear factor erythroid 2-related factor 2 (Nrf2)-antioxidant responsive element (ARE) pathway under oxidative stress [49], EAAC1 transport activity depends mainly on its cell surface expression, rather than *de novo* synthesis of the protein [48,50]. In the process of exocytosis, soluble *N*-ethylmaleimide-sensitive attachment protein receptor (SNARE) proteins facilitate the fusion of secretory vesicles to the plasma membrane. SNAP-23, a member of the SNARE family in the plasma membrane, is involved in EAAC1 trafficking to the plasma membrane [51]. The half-life of EAAC1 expression on the plasma membrane is approx. 5–7 min [48]. A substantial intracellular pool of EAAC1 is accomplished by endocytosis. Constitutive EAAC1 trafficking from endosome relies on a small GTP binding protein (G protein) activity. Small Rab GTPases regulate intracellular vesicle trafficking, especially in neurons [52,53]. These small G proteins are localized at the cytoplasmic faces of distinct membrane compartments. Recent studies demonstrated that EAAC1 was recycled back from endosomes to the plasma membrane in a Rab11-dependent manner [54] and that Rab11 dysfunction slowed EAAC1 trafficking to the cell surface, leading to impairments of both cysteine uptake and GSH synthesis [55].

### 4.4. GTRAP3-18

EAAC1 expression on the plasma membrane is negatively regulated by glutamate transport associated protein 3-18 (GTRAP3-18). By a yeast two-hybrid screen system from rat brain, GTRAP3-18 was isolated as an EAAC1-interacting protein [56]. GTRAP3-18 can specifically interact with EAAC1, but not with the other types of glutamate transporters. GTRAP3-18 is a member of the prenylated Rab acceptor (PRA) family [57]. There are two isoforms, PRA1 and PRA2, showing intracellular localization to the Golgi complex and the endoplasmic reticulum (ER), respectively [57]. Mouse GTRAP3-18 is almost identical to rat PRA2 [57,58]. GTRAP3-18/PRA2 is a 188-amino-acid protein and localizes in the ER with two extensive hydrophobic domains spanning amino acids 47 to 82 and 101 to 135, respectively [57], which are tightly attached to the ER membrane with four transmembrane domains and cytosolic *N* and *C* termini [59]. GTRAP3-18 tends to be dimeric [56], and the oligomerization of the four transmembrane domains is crucial for its activity [59]. Similarly, the oligomeric nature of the Yip family (the PRA family in yeast) has been demonstrated to enhance the protein-binding activity and selectivity [60].

The homo-oligomerization of GTRAP3-18 molecules may enhance its activity and specificity to certain oligomeric protein complexes. The C-terminal of GTRAP3-18 has a cluster of basic residues and a weak coiled-coil formation, which mediates protein-protein interaction. Changing the basic residues to an acidic residue (glutamate) resulted in partial localization of GTRAP3-18 to the Golgi complex [57]. This finding suggests that the *C*-terminal domain of GTRAP3-18 determines the intracellular localization to the ER. The hydrophobic domain interaction between GTRAP3-18 and EAAC1 may inhibit EAAC1’s exit from the ER [59]. GTRAP3-18 can bind to the *C*-terninal domain of EAAC1 (the last 87 amino acids) [56]. It can keep EAAC1 in the ER to inhibit EAAC1-mediated glutamate uptake, and it was observed that the glutamate uptake was elevated in accord with the reduction of the GTRAP3-18 protein level [56]. GTRAP3-18 protein with truncation of the *C*-terminal domain was unable to reduce ER-Golgi trafficking of EAAC1, whereas *N*-terminal truncation of GTRAP3-18 retained this ability [59]. GTRAP3-18 contains two PKC phosphorylation sites as well as two putative cAMP-dependent protein kinase and calcium/calmodulin-dependent protein kinase II phosphorylation sites [58]. There are two serine residues (S18 and S30) and three tyrosine residues (Y42, Y47 and Y182) in mouse GTRAP3-18 sequences. GTRAP3-18 may be regulated by protein phosphorylation via intracellular signaling molecules.

ADP-ribosylation factor-like 6 interacting protein 1 (Arl6ip1) is a GTRAP3-18-associated protein to act as a positive modulator of EAAC1-mediated glutamate transport in a PKC activity-dependent manner [61] (Figure 2). Arl6ip1 can interact with the hydrophobic region (amino acids 103–117) of GTRAP3-18 and decrease the number of GTRAP3-18 molecules available for interacting with EAAC1 [61]. Arl6ip1 cannot directly interact with EAAC1; however, it indirectly promotes EAAC1-mediated glutamate transport activity. Arl6ip1 also has PKC phosphorylation motifs [61]. PKC activation might increase GTRAP3-18/Arl6ip1 interaction, while decreasing GTRAP3-18/EAAC1 interaction in neurons [61].

### 4.5. Possible Regulation of ER-Golgi Transport by GTRAP3-18

Protein prenylation on *C*-terminal cysteine (Cys) residues plays an important role in its molecular function. Most Rab proteins have either a Cys-x-Cys or Cys-Cys motif, of which both Cys residues are subject to prenylation (geranylgeranylation) [62]. This process is required not only for the membrane association but also for the protein-protein interaction [63,64]. GTRAP3-18 binds to Rab1A and Rab3A in either the GDP- (inactive) or GTP-bound (active) state, but the interaction was abolished when the Cys-Cys prenylation motif of Rab was deleted *in vitro* [57]. This result suggests that the prenylation might be needed for the interaction between GTRAP3-18 and these Rabs.

The function of Rab1A is to promote ER-Golgi transport of vesicles. A recent study demonstrated that GTRAP3-18, most likely due to its Rab1 inhibitory action, inhibits neurite growth *in vitro* [65]. Rab3A is the most abundant Rab protein, localized to synaptic vesicles [64] to play a role in the recruitment of synaptic vesicles for exocytosis. Rab3A plays a key regulatory role in Ca^2+^-dependent exocytosis, particularly in neurotransmitter release from nerve terminals [66]. Although GTRAP3-18 is thought to be an ER protein, some reports have showed the subcellular localization of GTRAP3-18 in both the cytosol and the cell surface *in vitro* [56,67], suggesting a possible regulatory function for Rab3A.

### 4.6. Physiological Roles of GTRAP3-18

The physiological role of GTRAP3-18 is still poorly understood. The basal level of GTRAP3-18 is essentially low, and it is up-regulated by cell differentiation, heat shock, and oxidative stress [68–70]. Chronic administration of morphine to mice leads to a 300% to 400% increase in the GTRAP3-18 mRNA level in the amygdala [71]. Chronic morphine treatment also increased δ-opioid receptor expression in the nucleus accumbens [72]. GTRAP3-18 knockdown significantly decreased the withdrawal response to chronic morphine treatment [72], although the precise mechanism of GTRAP3-18 in the withdrawal responses to morphine is still elusive. In addition, GTRAP3-18 is reported to be able to regulate the trafficking of other transporters and receptors, such as dopamine transporter, GABA transporter (GAT-1), β_2_-adrenergic receptor, α_1_β receptor, and dopamine D_2_ receptor [59]. The common feature of these transporters and receptors is the formation of oligomeric complex before their exit from the ER. GTRAP3-18 may universally control neurotransmission by regulating the ER-Golgi transport of neuronal transporters and receptors.

### 4.7. Induction of GTRAP3-18 by Methyl-β-cyclodextrin

In the brain, GTRAP3-18 is widely expressed in the cerebral cortex, striatum, hippocampus and cerebellum [58]. GTRAP3-18 distribution in the brain showed widespread expression colocalized to neurons [56]. Strong GTRAP3-18 immunoreactivity was observed in the neuron-rich stratum pyramidale of the hippocampus and the Purkinje cells of the cerebellum [58], consistent with the distribution of EAAC1 [73–75]. In an *in vivo* study, the intraventricular administration of GTRAP3-18 antisense oligomers significantly increased cortical glutamate uptake by EAAC1 [56]. Methyl-β-cyclodextrin (MeβCD) significantly reduced Na^+^-dependent EAAC1-mediated [^3^H]glutamate uptake and increased GTRAP3-18 protein expression *in vitro* [76]. Intracerebroventricular administration of MeβCD to the mouse brain resulted in a significant increase in GTRAP3-18 immunoreactivity in the hippocampus and cerebral cortex [76].

Cholesterol is required for Na^+^-dependent glutamate transport [77]. MeβCD has a high affinity for cholesterol and has been shown to promote the efflux of cholesterol from cells [78]. Cholesterol depletion by MeβCD has been shown to induce cAMP response element-mediated gene expression [79] and to increase the activity of the extracellular signal-related kinase (ERK) [80]. cAMP- and ERK-dependent transcription factors may be responsible for the MeβCD-induced increase in GTRAP3-18 expression.

### 4.8. Regulation of GSH Synthesis by GTRAP3-18

Our previous cell culture studies demonstrated that increased GTRAP3-18 expression by MeβCD decreased the intracellular GSH content and showed vulnerability to oxidative stress, whereas knockdown of GTRAP3-18 expression by antisense oligonucleotide increased the intracellular GSH content and showed resistance to oxidative stress [67,81]. Intracerebroventricular injection models with MeβCD and siRNA for GTRAP3-18 also showed that increased or decreased GTRAP3-18 expression resulted in decreased or increased GSH level in the hippocampus, respectively. GTRAP3-18 negatively regulates the neuronal GSH level by direct interaction with EAAC1, which mediates neuronal cysteine uptake [67,81,82]. Our results suggest that GTRAP3-18 is a potential target for increasing the neuronal GSH level.

### 4.9. GTRAP3-18-Deficient Mouse

To further investigate the potential regulatory mechanism(s) underlying the increase in the neuronal GSH level *in vivo*, we generated GTRAP3-18-deficient (GTRAP3-18−/−) mice using embryonic stem cell technology [82]. The distribution of genotypes was close to the expected Mendelian frequency. GTRAP3-18−/− mice showed neither hindlimb clasping behavior, which is observed in neurodegenerative mice, nor histological abnormality in the brain. A recent study showed that the expression of GTRAP3-18 significantly declined from the embryonic stage to post-natal stage in the rat brains [65]. In addition, *in vitro* studies demonstrated that overexpression of GTRAP3-18 inhibited neurite outgrowth by blocking Rab1 function, whereas the knockdown of GTRAP3-18 had no effect on neurite length or the cell viability [65]. These results suggest a minor influence of the deletion of *GTRAP3-18* on neurodevelopment, at least morphologically.

The GTRAP3-18−/− mice showed an increased expression of EAAC1 on the plasma membrane [82]. EAAC1 activity is thought to largely depend on its cell surface expression, and not on *de novo* protein synthesis. The total amounts of brain glutamate, glycine, and GABA did not differ significantly between GTRAP3-18−/− and wild-type mice, but those of cysteine and GSH in the brain were significantly higher in the GTRAP3-18−/− mice compared to the wild-type mice. These results indicate that the ability of EAAC1 to increase cysteine uptake for GSH synthesis is potentiated in GTRAP3-18−/− mice. An immunostaining analysis showed that the increased GSH level was derived from neurons, but not from astrocytes or microglias [82]. Specifically, brain slices from GTRAP3-18−/− mice were resistant to oxidative stress. These results suggest that inhibition of GTRAP3-18 function leads to neuroprotection by increasing neuronal GSH synthesis.

Previous studies demonstrated that EAAC1 expression was increased in the rat hippocampus during the Morris water maze [83] and contextual fear conditioning tests, or by induction of long-term potentiation (LTP) in the CA1 region [84], while it was reported that EAAC1−/− mice showed impaired learning and memory [85,86]. Glutamate uptake is involved in memory formation [87–89]. Glial glutamate transporters can affect the duration and amplitude of excitatory postsynaptic potentials and currents by glutamate uptake [90], whereas the contribution of EAAC1 to the synaptic clearance of glutamate seems to be negligible [38]. Thus, it is unlikely that increased EAAC1 expression on the plasma membrane in GTRAP3-18−/− mice facilitates LTP induction by increasing the synaptic clearance of glutamate.

Another possible mechanism underlying the cognitive decline in EAAC1−/− mice is mediated by neuronal GSH depletion with aging. The presenile, but not young, EAAC1−/− mice showed brain atrophy and cognitive decline with aging compared to age-matched wild-type mice [85]. Neurons in the hippocampus of the EAAC1−/− mice showed low GSH and high oxidant levels, causing them to be vulnerable to oxidative stress. GSH depletion due to EAAC1 dysfunction could be the primary cause of age-dependent neurodegeneration. In our study [82], GTRAP3-18−/− mice showed high neuronal GSH levels and resistance to oxidative stress. They also showed better performance at forced motor/spatial learning and memory tests using rotarod and Morris water maze tests compared to age-matched wild-type mice. However, all of the mice in this study were under 6 months old, which is not old enough to develop age-dependent neurodegeneration. It is still unclear how GTRAP3-18 regulates the hippocampus-dependent learning process. The possibility that GTRAP3-18−/− mice might have facilitated learning and memory functions due to mechanisms not related to EAAC1 cannot be excluded.

## 5. Antioxidant Supplementation

Brain accessible antioxidants may provide a potential approach to slow the onset and progression of neurodegenerative diseases. However, approaches for increasing the GSH level in the brain are a matter of dispute. At present, there is no exogenous antioxidant for clinical use to demonstrate a therapeutic effect against neurodegeneration.

### 5.1. l-cysteine and Glutathione

l-cysteine barely penetrates the blood–brain barrier (BBB) due to its lack of an acidic omega side chain [36]. GSH also does not cross the BBB easily, and it is rapidly oxidized into GSSG in blood [91–93]. The plasma half-life of intravenously administered GSH is approximate 2–3 min [93–95] and only 0.5% of injected GSH could pass the BBB *in vivo* [91]. Peripherally administered GSH does not seem to function as an antioxidant in the brain.

### 5.2. Vitamins

Ascorbate (Vitamin C) and α-tocopherol (Vitamin E) are also important antioxidants in the brain [96–98]. The concentration of ascorbate in the human brain ranges from 1 to 2.6 mM [99], similar to that the concentration of GSH in the brain. However, humans cannot produce ascorbate and the BBB almost completely blocks ascorbate penetration into the brain [100]. Alpha-tocopherol is the most potent antioxidant in the lipid part of the biological membrane [98]. However, the α-tocopherol level in the brain is relatively lower than those of ascorbate and GSH [17,98,99,101]. In addition, the oral administration of α-tocopherol did not increase its concentration in the central nervous system (CNS) due to its limited penetration of the BBB [102].

Indeed, supplementation with ascorbate, α-tocopherol, or both, have failed to produce conspicuous benefit in patients with neurodegenerative diseases such as PD and Alzheimer’s disease (AD) [103–107], although several conflicting lines of evidence have been reported [108–110]. In addition, it was reported that the levels of ascorbate and α-tocopherol in the CNS did not change in patients with PD and AD, compared with controls [27,28,101,111]. The principal function of ascorbate and α-tocopherol as antioxidants may be based on a synergistic effect with GSH in the CNS [97,112].

### 5.3. Uric Acid

Uric acid (UA) is an important antioxidant in blood. UA scavenges singlet oxygen, hydroxyl radicals, and peroxynitrite in blood at its physiological concentration [113]. Indeed, epidemiological studies have shown an association between low plasma UA levels and neurodegenerative diseases [114]. However, the UA level is much lower in the CNS than in blood [115,116]. It is still unclear whether UA is able to act as a direct antioxidant in the brain. We recently reported that UA treatment increased both cysteine uptake *in vitro* and the brain GSH level at the physiological concentration *in vivo* [117]. Our results also showed that mouse hippocampal slices treated with UA were resistant to oxidative stress and increased the GSH level in neurons. UA might indirectly exert a neuroprotective effect via the mediation of GSH synthesis in the brain by EAAC1, although further studies are needed to elucidate the mechanisms of any UA-mediated neuroprotective effect in the brain.

### 5.4. N-acetylcysteine

*N*-acetylcysteine (NAC) is a promising compound to increase GSH synthesis in the brain. NAC stimulates GSH synthesis not only by providing a source of cysteine, but also by activating GR [118]. NAC can permeate the cell membrane without the neuronal cysteine transporter EAAC1 [85,119] to yield cysteine via intracellular deacetylation. NAC also acts as a direct chemical antioxidant, although its potency is less than that of GSH [120]. Chronic dietary administration with 0.3% NAC containing food pellets reduced the protein carbonyl contents in synaptic mitochondria even without affecting the GSH level [121], although a single intraperitoneal injection of 150 mg/kg NAC can increase the GSH level in the mouse brain [85].

Indeed, neuroprotective effects of NAC have been reported on amyloid β [122], 1-methyl-4-phenyl-1,2,3,6-tetrahydropyridine (MPTP) [123], 6-hydroxydopamine [124], CNS trauma [125], and brain ischemia-induced neurotoxicity [119,126]. We also confirmed the neuroprotective ability of NAC to increase the GSH level in mesencephalic dopaminergic neurons [127]. However, the effect of NAC is not neuron-specific [128], and excessive production of cysteine may be neurotoxic [33–36]. The appropriate dose of NAC for clinical use against neurodegeneration remains to be determined.

## 6. Conclusions

In the treatment of neurodegenerative diseases, replacement therapy is still the main approach to ameliorate the symptoms, but not to inhibit neurodegeneration, whereas antioxidant therapy has been suggested to be a fundamental approach against neurodegeneration. Among the antioxidant agents, GSH plays a critical role in the CNS. Increasing the neuronal GSH content in the brain is a promising approach by which neuroprotective effects have been demonstrated. Not a small number of published papers have suggested potential candidates to increase neuronal GSH content. However, most of these candidates simply act as precursors for cysteine or GSH. Considering delivery systems into the brain, these exogenous supplementations do not seem to be the best way to increase the neuronal GSH level. We have focused on GTRAP3-18 as a target for neuroprotection. GTRAP3-18 is localized in neurons to negatively regulate EAAC1 function, a mechanism by which neurons can increase GSH synthesis. The endogenous regulation of GTRAP3-18 would be an alternative approach to the inhibition of neurodegeneration. However, considering a potential ability to regulate intracellular trafficking of various receptors and transporters, other, as yet unknown, physiological functions of GTRAP3-18 *in vivo* should be clarified.
